# *Moringa oleifera* Seeds Characterization and Potential Uses as Food

**DOI:** 10.3390/foods11111629

**Published:** 2022-05-31

**Authors:** Adèle Gautier, Carla Margarida Duarte, Isabel Sousa

**Affiliations:** LEAF—Linking Landscape, Environment, Agriculture and Food, Higher Institute of Agronomy, Universidade de Lisboa, Tapada da Ajuda, 1349-017 Lisboa, Portugal; adele-gautier@outlook.fr (A.G.); carladuarte@isa.ulisboa.pt (C.M.D.)

**Keywords:** *Moringa oleifera* seed beverage, yoghurt-like, lactic acid fermentation, rheology

## Abstract

Despite the fact *Moringa oleifera* (MO)-based foods present a very good and nutritionally well-balanced composition, they face some issues related to seed bitterness, which is the most challenging barrier to consumer acceptance. Different processing methods were tested to produce MO toasted seeds, MO-based beverage, and yoghurt-like products which were chemically and rheologically analyzed. The protein content ranged from 3.68% in the beverage, to 14.73% in the yoghurt and 40.21% in MO toasted seeds. A totally debittered beverage could not be accomplished, but the MO yoghurt-like showed a very nice flavor. Nutrition claims for minerals in toasted seeds could be considered for magnesium, phosphorus, iron, copper, zinc, and manganese, which confirms the *M. oleifera* seed richness in several minerals. The MO beverage presented less extended shear-thinning behavior (17.4 Pa·s) than commercial vegetable beverages and two pulse-based beverages developed in a previous study. The MO yoghurt-like product showed a gel structure similar to the dairy yoghurt, making it a promising new plant-based alternative. Further work must be performed in the future to debitter more efficiently the raw seeds to achieve a more pleasant MO-based beverage. The developed MO seed-based products may settle another font of high protein plant-based food.

## 1. Introduction

*Moringa oleifera* (MO) is considered the most nutrient-rich plant on earth and is recognized for its vast therapeutic properties since ancient times. The different parts of the plant are used across the African continent for a variety of medicinal, nutritional, phytochemical and horticultural purposes. The folk medicinal uses of *M. oleifera* leaves are attributed to the presence of functional bioactive compounds, such as phenolic acids, flavonoids, alkaloids, phytosterols, natural sugars, vitamins, minerals, and organic acids [1,2]. MO leaves provides 7 times more vitamin C than oranges, 10 times more vitamin A than carrots, 17 times more calcium than milk, 9 times more protein than yoghurt, 15 times more potassium than bananas, and 25 times more iron than spinach [3]. It is very adequate for biofortification in terms of minerals (which represents one of the greatest deficiencies in African diets). MO leaves have been consumed by Asian people for millennia as a healthy food product and is the most common way of using MO in culinary. In recent years, the various food applications of the leaf powder (e.g., bread, yoghurt, biscuits) have led to an interesting uprising market in developed countries. This commercially well succeeded use of Moringa leaves the seeds as by-products.

*M. oleifera* seeds are a promising alternative for food supplementation using. The use of *M. oleifera* seed flour as wheat flour fortifying in bread, biscuits and cookies production [4], and also the seed extract as thickening agent in milk yoghurt production [5] are known to improve food nutritional and rheological qualities. Several in vitro studies have also shown that *M. oleifera* seed possess anti-inflammatory and immunomodulatory activities, antimicrobial and anticancer potential, and antioxidant activity [6,7]. In Guinea-Bissau, the MO seeds are used to regulate blood pressure and treat asthma [8], but across Africa they are also used to treat diabetes, fever, malaria, and sexual dysfunction [9]. The MO seed composition presents higher content in protein (36%), fat (38.7%), vitamin E (752 mg per 100 g of dry seed), magnesium (635 mg per 100 g of dry seed) and copper (5.2 mg per 100 g of dry seed), when compared to MO leaves and pods [10]. The fried MO seeds are eaten in Nigeria and are said to taste similar to groundnuts. The seeds are added locally to sauces for their bitter taste. Research shows that MO seed oil contains around 76% PUFA (linoleic acid, linolenic acid, and oleic acid) [11], which have the ability to control cholesterol, making it ideal for use as a substitute for olive oil [12] and give it a high oxidative stability. The extraction of this oil is a promising activity since its use is welcomed in cosmetics as neutral carriage for fragrance, soaps, as a lubricant, with increasing market price.

Most plants lose their nutritive properties when processed. *M. oleifera* seeds contain a few bitter peptides with some hemagglutinating activity, glucosinolates (65.5 μmol/g seed, which equates to approximately 40 mg/g or 4% of the seed weight) [13] and phytates. All glucosinolates are strongly bitter compounds. They can be extracted with mixtures of water and alcohol due to its solubility in water, and there are some heat labile [14]. Phytates are present to an extent of 1% to 6% and may reduce mineral bioavailability in monogastric animals, particularly, Zn^2+^ and Ca^2+^ [15]. The presence of phytate and other anti-nutrients can reduce the bioavailability of certain nutrients, but fermentation can be used to neutralize this effect and potentiate maximum utilization of the required nutrients from the seeds. When compared, the nutritive content of raw, germinated and fermented MO seed flour, it was found that phytochemicals were higher in raw seed flour and amino acid content was at its peak in fermented and germinated seed flour [16,17]. This can be a result of the biochemical changes during fermentation which include increasing the protein content, essential amino acid and polyunsaturated fatty acid profiles, and reduce anti-nutrient compositions of MO seed, being more efficient than germination processing techniques [17]. Thus, the *M. oleifera* seed excellent nutritional properties and its low toxicity after efficient thermal treatment, provides a good alternative to some pulse seeds (poor in sulphur containing amino acids) as a source of high-quality protein, oil, and antioxidant compounds [15].

This work started with chemical characterization of *M. oleifera* seeds to support its future use as food. Different processing techniques such as soaking, toasting, cooking and fermentation were tested and discussed for the development of MO seeds-based foods, to achieve good sensory features and remove the bitter taste. The flow behavior of the developed MO yoghurt-like was studied and compared to commercial soy yoghurt and low-fat dairy yoghurt, and the MO-based beverage was compared to chickpea- and lupin-based beverages developed in a previous study [18].

## 2. Materials and Methods

Different processing techniques were tested for the development of *Moringa oleifera* seeds-based foods to achieve good sensory features and remove the bitter taste: soaking, cooking, toasting at different time and temperature, and also using lactic acid fermentation. Eventually, three different processes and derived foods were chosen according to the literature review aiming at a good sensory quality: *M. oleifera* toasted seeds, MO based beverage, and a MO yoghurt-like product.

### 2.1. M. oleifera Toasted Seed Preparation

*Moringa oleifera* dehulled seeds (kernels) were used from an Indian supplier (Ramamoorthy Exports).

The toasted seed production evolved into the following final optimization: 20 g of dried seeds were soaked twice in warm tap water (30–35 °C) and once in cold tap water (15–20 °C) in a proportion of 1:3 (*w/v*) for ca. 16 h [19]. All soaking waters were discarded. Then, the soaked seeds were toasted in a forced air oven at 150 °C for 30 min.

Both raw and toasted MO seeds were stored at room temperature for further analysis.

### 2.2. M. oleifera-Based Beverage Preparation

**Figure 1:**
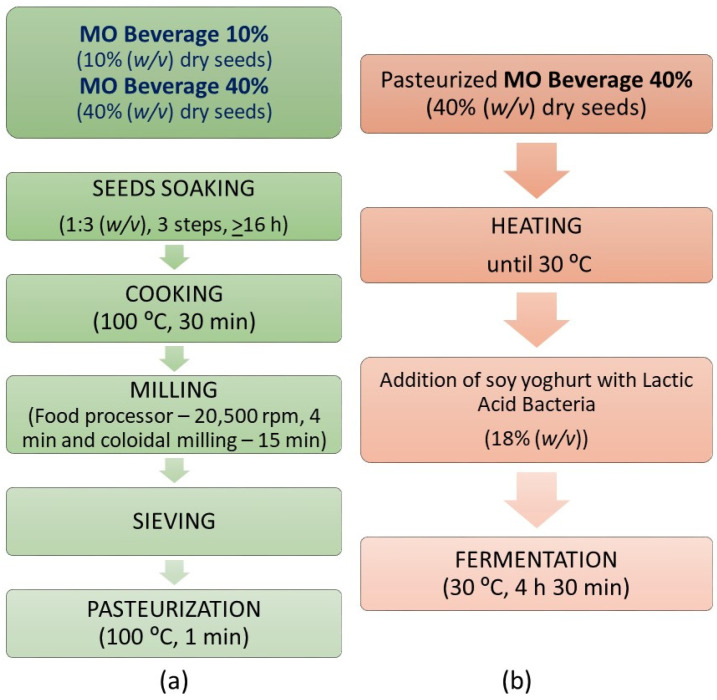
(**a**) *M. oleifera* beverage and (**b**) yoghurt-like manufacturing procedures.

For MO-based beverage and yoghurt-like production two different seed concentrations were used: 10% and 40% (*w/v*) of total dry seeds, respectively. The seeds were soaked as described previously, cooked in boiling water for 30 min in a pressure pan [20] and liquids discarded. The cooked seeds were drained, and the corresponding volume of fresh tap water was added to achieve 10% or 40% (*w/v*) of dry seeds in water. Then, the mixture was milled in the food processor (Bimby-Worwerk, Wuppertal, Germany) at 20,500 rpm for 4 min with only 250 mL of fresh tap water (adapted from previous works [21,22]), followed by colloidal milling performed by a mortar grinder (Pulverisette 2, Fritsch GmbH, Idar-Oberstein, Germany) at a lab scale, at 70 rpm, for 15 min using the remaining volume of water. The resulting beverage was sieved with a strainer before being bottled in sterilized flasks. In the pasteurization step, adapted from a previous work [21], the capsulated filled flasks (beverage temperature above 90 °C), were submitted to a thermal shock, inside the pressure cooker for 1 min, in boiling water (Figure 1a).

After production, both beverage concentrations were stored at 4 °C for a maximum of 7 days for further analysis or fermentation.

### 2.3. M. oleifera Yoghurt-like Preparation

A commercial soy yoghurt (Alpro natural 125 g, Danone, Portugal) containing the starter cultures (*S. thermophilus* and *L. bulgaricus*) was added in a proportion of 18% (*w/v*) to the corresponding volume of refrigerated MO-based beverage with 40% (*w/v*) of dry seeds, for further lactic fermentation (Figure 1b). The preparation was then thoroughly homogenized during the heating stage and was incubated at 30–32 °C during 4 h and 30 min. After production, the MO yoghurt-like were stored at 4 °C for a maximum of 7 days for physico-chemical analyses.

### 2.4. Physico-Chemical Analysis

The water activity (aw) was determined in Hygrolab equipment (Rotronic, Hauppauge, NY, USA) at 20 ± 1 °C on triplicates of raw and toasted *M. oleifera* seed flours.

The total protein determination of MO raw seeds was performed by Dumas Nitrogen Analyser NDA 702 (Velp Scientifica, Usmate, Italy) and the correction factor used to convert nitrogen into crude protein was 6.25 [17]. All analyses were carried out in triplicate and expressed as a percentage.

Total lipid analysis of MO seeds and yoghurt-like were based on the Soxhlet fat extraction method and carried out in triplicates according to Ijarotimi and coworkers method [17] with some modifications: 1 g sample was weighed into a thimble and covered with absorbent cotton, while about 50 mL of petroleum ether (100 °C) (ref 32299-2500, Honeywell, Charlotte, NC, USA) was added to a pre-weighed cup. Both thimble and cup were attached to the Extraction Unit (TecatorSoxtec, Model 2043, Hilleroed, Denmark). The soluble lipid in the samples was extracted into the solvent for 15 min and rinsed for 1 h and 30 min. The distilled solvent was then condensed and collected. A final drying step (105 °C for 7 h) evaporated the last traces of solvent from the extraction cups. The dried extraction cups were weighed and mass percentage of lipid content was calculated according to Equation (1):

Lipid (%) = [(Initial cup weight − Final cup weight)/(Weight of sample)] × 100(1)

The total lipid content of MO beverage was estimated using the Gerber method [23]. Briefly, a butyrometer was used where acid hydrolysis is carried out for the digestion of sample protein triplicates with sulphuric acid (ref 1082-00, Weber Scientific, Hamilton, NJ, USA) and use of amyl alcohol (ref 1087-00, Weber Scientific, Hamilton, NJ, USA), in a water bath for 5 min at 65 ± 2 °C, followed by separation of fat by centrifugation (1100 rpm for 4 min). The fat value was read directly on the butyrometer scale as a mass percentage of the fat content in the sample.

The total acidity of the MO beverage and yoghurt-like were analyzed in triplicates in accordance with adapted OIV-MA-AS313-01:R2015 international method [24], with some modifications. Briefly, each sample (20 mL) was diluted with 25 mL of boiled water. Then 3 droplets of phenolphthalein were added, and the titration was performed with 0.1 N NaOH (aq.) (ref 221465, Sigma-Aldrich, Merck, Darmstadt, Germany). Total Acidity was expressed in milliequivalents (mEq) of acid/L.

Dry matter of MO raw and toasted seeds, beverage and yoghurt-like was determined gravimetrically by drying at 105 ± 5 °C in a forced air oven (Binder, FED 115, Tuttlingen, Germany) until constant weight of the sample and its solid residue were calculated as percentage. The percentage of moisture in triplicates was calculated according to Equation (2):

Moisture (%) = 100 − [(W3 − W1)/(W2 − W1)] × 100(2)

where W1 is the weight of the crucible, W2 is the weight of the crucible and sample before drying at 110 °C, and W3 is the weight of the crucible with the sample residue after drying and cooling in airtight desiccators. 

Ash content was also determined gravimetrically by incineration at 550 ± 10 °C in a muffle furnace (Snol 164 LHM01, Utena, Lithuania) for *M. oleifera* beverage and yoghurt-like according to AOAC 923.03 (2005) [25], respectively. Results of triplicates are expressed as a percentage.

Carbohydrate content was estimated by the difference to 100% of main constituents (moisture, ash, protein and fat). The energy value of MO beverage and yoghurt-like, was calculated considering the conversion factors [26] for protein (4 kcal/g; 17 kJ/g), fat (9 kcal/g; 37 kJ/g) and carbohydrates (4 kcal/g; 17 kJ/g).

The mineral content in seeds, beverage and yoghurt-like were carried out in triplicates by inductively coupled plasma optical emission spectrometry (ICP-OES: iCAP 7000 Series Spectrometer equipped with ASX-520 AutoSampler, Thermo Scientific, Waltham, MA, USA). Briefly, 0.25–0.30 g of seed flour, 5–6 g of MO yoghurt-like and 5 mL of MO beverage were transferred to digestion vessels and respective volumes in a proportion of 3:1 of HCl (37%) (ref 30721, Fluka, Honeywell, Charlotte, NC, USA) and HNO_3_ (65%) (ref 695041, Sigma-Aldrich, Merck, Darmstadt, Germany), were added. The digestion (SCP Science, DigiPREP MS, Baie d’Urfe, QC, Canada) took place at 15 min/45 °C, 15 min/80 °C, and 60 min/105 °C. After cooling, distilled water was added up to 50 mL, and the solution was left to decant. Finally, the clear supernatant was used in ICP analysis. Eleven elements (Na, K, Ca, Mg, P, S, Fe, Cu, Zn, Mn, B) were determined in triplicate. Results are expressed as mg of mineral element per 100 g of sample (dry matter for toasted seeds and weight for yoghurt), except for beverages (mg/100 mL).

The color of the *M. oleifera* beverage and yoghurt-like was measured using a Minolta CR-300 (Tokyo, Japan) tristimulus colorimeter that was calibrated using a white standard porcelain plate (L* 96.96; a* 0.37; b* 2.10). The results were expressed in accordance with the CIELAB uniform color system with reference to standard illuminate D65 (average daylight conditions) and a visual angle of 2°. The color parameters determined were L*, which accounts for the lightness (i.e., 0% for black and 100% for white), a* ranges from green to red and b* from blue to yellow, which corresponds to a numerical variation from −60 to +60. The measurements were conducted at room temperature under similar light conditions (i.e., 50 mm^2^ measuring area per measurement) and replicated 6 times per sample. 

The total color difference between the samples was calculated according to the Equation (3). UHT whole cow milk and soy beverage, but also commercial cow yoghurt and soy yoghurt were used as references for MO beverage and MO yoghurt-like, respectively.

(3) $$\Delta E^{*}=\sqrt{{\left(\Delta L^{*}\right)}^{2}+{\left(\Delta a^{*}\right)}^{2}+{\left(\Delta b^{*}\right)}^{2}}$$

Considering that if ΔE* is higher than five units, the color difference is detectable by the human eye [27].

The pH (pH meter Basic 20, CRISON Instruments, Barcelona, Spain) of the different MO beverage and yoghurt-like was measured at room temperature during the day after production.

### 2.5. Sensory Evaluation of Samples

The development of the MO seed-based food prototypes also included a concise evaluation of its sensory features, after each new processing step condition [28]. This evaluation was performed by the authors and other people in the lab (unrepresentative sample), to estimate the consumer’s perception of each prototype, and to support the decision for the next processing step.

### 2.6. Rheological Measurements

The shear viscosity of the *M. oleifera* seed-based beverage and yoghurt-like, commercial soy yoghurt and low-fat dairy yoghurt (Mimosa magro 125 g, Lactogal, Portugal) was measured using a controlled-stress rheometer (Thermo Fisher Scientific, Haake MARS III, Karlsruhe, Germany), with a CCB/CC25 DIN Ti concentric cylinder geometry to avoid phase separation of the beverage, and with the lower plate TMP60 (222-1891) and cone DC60/2° Ti (222-1932) for the yoghurt. The measurements for yoghurt were carried out at 20 ± 1 °C and at 8 ± 1 °C, temperature at which yoghurts are usually consumed [29]. The measurements for beverage were carried out at 20 ± 1 °C. The steady shear viscosity measurements were performed with shear rates from 1.0 × 10^−5^ to 1.0 × 10^3^ s^−1^. Tests took 21 min each and were performed in triplicate with well homogenized yoghurt and beverage. The flow curves were fitted to the Carreau model (Equation (4)), since the yoghurt and beverage are non-Newtonian fluids and evidenced shear-thinning behavior (that is, the viscosity decreases as the shear rate increases) [30]:

(4) $$\eta =\eta_{\infty }+\frac{\eta_{0}-\eta_{\infty }}{{\left(1+{\left(K\dot{\gamma }\right)}^{2}\right)}^{\frac{m}{2}}}$$

where “η_0_” is the first limiting (“zero” shear rate) Newtonian viscosity (Pa·s); η_∞_ is the second limiting (“infinite” shear rate) Newtonian viscosity (Pa·s); “$\dot{\gamma }$” is the shear rate (s^−1^); “K” is the relaxation time (s) and the reciprocal, 1/K ($\dot{\gamma }$c), is related to the critical shear rate (i.e., onset shear rate for shear-thinning); and “m” is the dimensionless constant related to power law and accounting for the deviation from the Newtonian behavior. The TRIOS Software (TA Instruments, v4.5.1.) was used for the data analysis.

All of the rheological tests were repeated at least three times. Yoghurt samples were allowed to rest for 300 s after placing it in the measuring device and covered with a layer of paraffin oil to prevent moisture loss.

### 2.7. Statistical Analysis

Analysis of variance (one-way ANOVA) was used to assess significant differences between samples at a significance level of 95% (*p* ≤ 0.05). Multiple comparisons were performed by Tukey HSD test. All statistical treatments were performed using SPSS Statistics (v.20, IBM SPSS Statistics, New York, NY, USA).

## 3. Results and Discussion

### 3.1. Progression on the Processing Techniques Used for the Development of Moringa oleifera Seed-Based Foods

The next Table 1 describes the main production steps tested for the development of *Moringa oleifera* seed-based foods to achieve good sensory features and remove the bitter taste of the seed.

### 3.2. Physico-Chemical Parameters of the Optimized M. oleifera Seed-Based Foods

The *M. oleifera* raw and toasted seeds did not present significant differences (Table 2) in the physico-chemical analysis, except for the protein content which is due to the significant moisture loss of the seed during toasting (0.33%), leading to a higher value (40.21%). The fat (37.2% (*w/w*)) and protein contents (36.8% (*w/w*)) obtained from raw seeds were similar to the ones mentioned in Gopalakrishnan’s work [10] (38.7% (*w/w*) and 36.0% (*w/w*), respectively) and inside the ranges presented by Brilhante and coworkers [6] for both parameters (fat: 30.8–41.2%; protein: 29.4–38.3%). The variation in these parameters can be attributed to the region where MO is planted and the growing conditions of the plant [6].

The comparison between fat and protein contents of MO toasted seed and toasted groundnuts, showed that the first one is nutritionally better. The MO toasted seed presented a lower fat content (34.95% vs. 49.6%) but higher protein value (40.21% vs. 25.6%) (Table 2) when compared to toasted groundnuts [31].

Raw and toasted MO seeds are relatively dry (4.74 and 0.33%, respectively) and their water activity allows to predict the seed stability and safety in terms of microbial growth. The lowest aw at which the majority of food spoilage occurs is 0.90, although some yeasts and molds may grow above 0.61 [32]. Both water activities of raw and toasted seeds (0.56 and 0.48, respectively) confirm their good shelf life capacity (Table 2).

The similar total acidity of raw and toasted seeds (3.7% and 3.5%) may be due to some bitter compounds of the MO seeds that were not extracted during the soaking step.

The *M. oleifera*-based beverage presented a high protein content (3.68% (*w/v*)) (Table 3)) when compared to cow milk (3.3–3.5%) and commercial non-dairy beverages (<1%) [18], being a good vegetable alternative to dairy protein like the chickpea beverage (3.24% *w/v*) and the lupin beverage (4.05% *w/v*) [33]. In total mitigation of the slight bitterness of MO beverage, a toasting step could be considered as a future process optimization, between cooking the seeds and the colloidal milling. In Ogunsina’s work [34], the MO flour used in bread and cookies fabrication was successfully debittered by heating the cooked kernels at 80 °C for 8 h.

Comparing pH values of the MO beverage (Table 3) with those obtained for pulse-based beverages in a previous study [18], there is some similarity with the acidic lupin beverages (pH < 6.0), and less with the chickpea beverages (pH = 6.7–7.2).

The acidification during fermentation was achieved (46.7 mEq/L) and was confirmed by its lower pH (5.45) when compared to the beverage, showing the acid lactic bacteria activity [35]. As expected, the low carbohydrate content (around 1.76%) of the MO beverage containing 40% (*w/v*) of dry seeds, was consumed by lactic acid bacteria during fermentation [35], which explain why the MO yoghurt-like had no carbohydrates (Table 3). In a different study [5], the addition of ultra filtered *M. oleifera* seed extract to dairy yoghurts, as thickening agent, showed that the MO seed enhanced the metabolic ability of lactic acid bacteria through its organic acids, phenolic acids and flavonoids, causing an accelerated drop in pH and the increase of the acidity of fortified dairy yogurts compared to the control samples. The dairy yoghurts fortified with MO extract [5] showed similar contents of fat (5.97–6.32 vs. 7.25), ashes (0.95–0.98 vs. 0.75) and moisture (77.38–77.89 vs. 81.10) to those obtained in this work, but lower content of protein (5.55–5.76 vs. 14.73), as MO extract was at lower proportion.

The higher content of fat, ashes, protein and energy value in MO yoghurt-like when compared to the MO beverage (Table 3), are due to the composition of its nutritional source (beverage with 40% *w/v* of dry seeds) which is four times richer than MO beverage (10% *w/v*). Adding to that, the relevant protein content in the MO yoghurt-like (14.73%), when compared to soy and dairy yoghurts (4–5%), settles down a new potential font of high protein plant-based food, highly competitive in the current commercial non-dairy markets.

The lesser moisture content of 81.1% in MO yoghurt is explained by 40% of MO seeds, responsible by the “creamy” consistency of the beverage, comparing to the beverage with only 10% of MO seeds.

The color of all samples (data not shown) differs from the references (∆E* > 12), meaning that its color is visibly distinguished by the human eye [27].

The purpose of food fortification involves the addition of essential nutrients such as vitamins and minerals to staple foods to improve their nutritional value, however, this work aimed at using the MO seed itself as a food product, so its nutritional composition comes only from the seed submitted to the processing conditions.

Some differences were found among the minerals in comparison to the few available literature on raw *M. oleifera* seeds composition [6,10,17]. These variations may be due to different methodologies and instruments used in the analysis (atomic absorption spectroscopy versus ICP-OES) along with M. oleifera different varieties edapho-climatic conditions, harvest period, and/or nutritional status of the plants, that influence the mineral contents of the seeds [36]. Nevertheless, the content of the following mineral elements described in Ijarotimi’s work [17] are very close to those found in this study for raw seeds (Table 4): calcium (128.33 mg/100 g), iron (7.33 mg/100 g), and copper (0.63 mg/100 g). On the contrary, the magnesium and sulphur contents found in MO raw seeds were significantly different in Gopalakrishnan’s work [10] (635 mg/100 g; 0.05 mg/100 g, respectively) when compared to the ones presented here (302.51 mg/100 g for magnesium and 1994.06 mg/100 g for sulphur).

In general, the toasted seeds presented a significant higher mineral content when compared to the raw ones (Table 4), explained by the effect of toasting with relevant moisture loss. Nutrition claims for minerals in toasted seeds could be considered as shown in Table 4 for magnesium, phosphorus, iron, copper, zinc and manganese, which confirms *M. oleifera* seed richness in several minerals. As shown, the mineral contribution of 100 g of toasted MO seed for attaining the dietary reference intakes (DRI) established for adults [26,37] was higher than the other two MO seed-based foods studied. Adding to that, all mineral elements shown in MO toasted seeds are higher than the ones present in toasted groundnut (e.g., Mg, 170 mg/100 g; P, 370 mg/100 g; Fe, 2.1 mg/100 g) [31].

As expected, both MO beverage and MO yoghurt-type presented lower mineral values when compared to the raw seeds (Table 4), which may be due to their leaching out during soaking and cooking processing steps. As known, the non-dairy commercial beverages and yoghurts usually show large discrepancy in nutrients compared to dairy products [38,39], conducting to potential nutritional deficiencies if not well balanced through the food diet [40]. Generally, the composition of commercial vegetable beverages and yoghurts comprises several additives, including minerals (especially calcium), to mimic the nutritional characteristics of dairy products [38,39], such as the commercial soy yoghurt used in this work, with 15% of calcium (equal to minimum DRI value) in its composition. Despite this, both MO beverage and yoghurt-like may have the nutrition claims for copper, zinc, and manganese.

### 3.3. Evaluation of the M. oleifera-Based Beverage and Yoghurt-like Rheology Behavior

**Figure 2:**
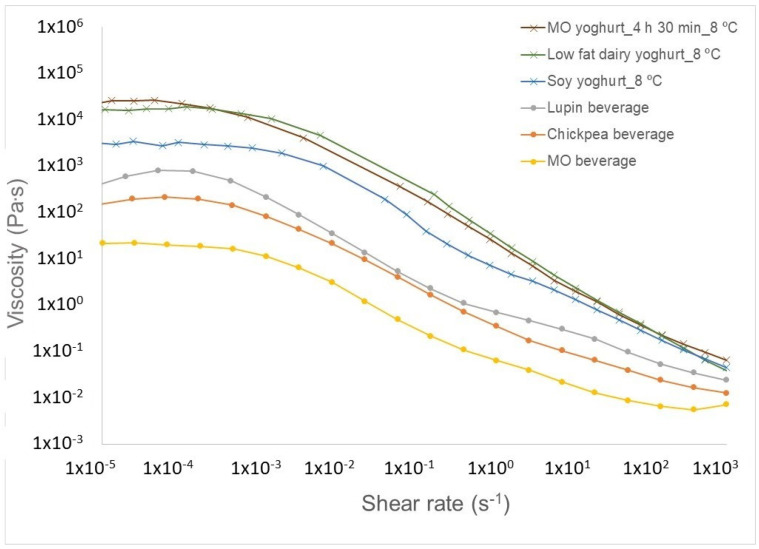
Flow curves showing the shear viscosity profile for the MO-based beverage and yoghurt-type compared to lupin- and chickpea-based beverages, and also to commercial soy and low fat dairy yoghurt, respectively.

The consistency of the *M. oleifera*-based dairy alternatives was compared through the steady shear flow behavior. In Figure 2 one can see the MO beverage compared to two pulse-based beverages developed previously by our research group, in a comparison to other current non-dairy alternative beverages. The MO-, chickpea- and lupin-based beverages presented a typical non-Newtonian shear-thinning behavior, very similar to the commercial hazelnut (η_0_ 125.4 Pa·s) and oat (η_0_ 52.6 Pa·s) beverages, this oat beverage being the consumer’s preference [18].

The MO-based beverage showed the lower shear-thinning behavior (η_0_ 17.4 Pa·s) when compared to the chickpea- or lupin-based ones (Figure 2 and Table 5). This is due to a weaker supramolecular structure more easily destroyed by the applied shear and the micro-scale structures within the fluid rearrange/align to facilitate shearing, reaching sooner a lower second Newtonian plateau. The MO beverage’s viscosity behavior was still far from the flow of chickpea beverage (17.4 against 176.4 Pa·s). As increasing MO concentration will impact negatively through the bitter taste, one possibility would be to use lactic fermentation in these beverages, to reduce bitterness and accommodate higher MO concentrations, giving high protein plant-based products.

The MO-based “yoghurt” displayed very similar flow curve to the low fat cow milk one, with a well-defined Newtonian plateau at low shear rates, followed by the shear-thinning region (Figure 2). The MO yoghurt incubated for 4 h 30 min presented the most extended shear-thinning behavior (η_0_ 21,465.7 against 3381.2 Pa·s) (Table 5) meaning that the internal structure in this yoghurt was stronger compared to soy yoghurt. This may be explained by its higher concentration in protein which allowed the gel structuring through the lactic acid bacteria production of exopolysaccharides and increase of protein links forming a strong network [41], with a lubricating effect from its relevant fat content which may have an increasing effect on the shear induced reduction of the viscosity [42].

## 4. Conclusions

The processing strategies adopted and the optimizations performed led to the development of *Moringa oleifera*-based foods such as toasted seeds, and plant-based alternatives to dairy products with several appealing features (e.g., protein and mineral contents, consistency) that may be potentially competitive in the current non-dairy plant-based market and also as a supplement to deficient diets on protein and minerals on the countries from where this crop is originated.

The higher protein content, lower fat concentration, and richness in minerals of the MO toasted seed, when compared to the toasted groundnuts, makes this oilyseed a nutricious and competitive snack.

The lactic acid fermentation of the MO beverage with 40% (*w/v*) of dry seeds showed a high protein-value product with a gel structure similar to the dairy yoghurt, making it a promising new plant-based alternative. Further work will be necessary to evaluate the texture of this gel on the basis of sensory characterization. 

Taking into account the MO beverage (10% (*w/v*) of dry seeds), further future optimizations must be performed, such as finding a process to debitter more efficiently the raw seeds to achieve a more pleasant MO-based beverage (e.g., toasting cooked kernels before milling, lactic fermentation and/or the addition of natural flavors which may improve the sensory perception). All of these aspects are being considered in ongoing and future work.

## Figures and Tables

**Table 1:** Progress of processing stages for *M. oleifera* seed-based foods (toasted seed, beverage and yoghurt-like) to achieve final optimization.

Task Description	Results/Sensory Evaluation	Further Steps	Decision Support
SEED TOASTING			
65 °C for 30 min	Still sour, but less than raw seed; sweet after flavor.	Increase toasting temperature to diminish or eliminate the sourness.	Control of temperature will help the removal of bitterness.
200 °C for 1 h 40 min + 260 °C for 16 min	Too much toasted, very bad taste (burnt).	Reduce toasting time.	
200 °C for 30 min	Seed without color change; still bitter and sweet.	Reduce toasting temperature.	
150 °C for 30 min	Too sweet, too bitter, not burnt.	Soak the kernels first.	
After soaking: 100 °C, for 30 min	Taste the same as raw, still too sweet and too bitter.	Keep soaking and increase the temperature to 150 °C.	
After soaking: 150 °C, for 30 min	Very crunchy, good taste (similar to toasted groundnuts).	Not necessary.	OK. Best performing procedure (BPP).
SEED SOAKING			
Soaking raw seeds (1:3 *w/v*). 3 stages: first two warm water (30–35 °C), last with cold water (15–20 °C) overnight.	Taste the same as the raw kernels, still too sour and too sweet.	Cook the seeds.	Soaking and cooking help to release the anti-nutrients, such as the bitter compounds, to the water.
Before cooking: Soaked seeds cooked in hot water (30 min, 100 °C)	The sweetness was lost, still a little bit of bitterness.	Addition of flavors to improve beverage taste.	OK. Sensory testing with new flavors.
SOAKED SEED MILLING			
Before cooking Food processor (4 min, 20,500 rpm) followed by Ultraturrax (1 min, 20,500 rpm) + addition of tap water to help milling efficiency and beverage homogenization.	Very strong bitter/raw taste. Sandy mouthfeel.	Cook the soaked seeds. To reduce sandy mouthfeel use the colloidal mill.	
After cooking Food processor (4 min, 20,500 rpm) followed by colloidal milling (70 rpm, 15 min) + addition of tap water to help milling efficiency and beverage homogenization.	Smooth and pleasant taste, with a slight bitterness on final beverage.	Not necessary.	OK. BPP.
FERMENTATION			
MO “cream”—MO beverage with 40% (*w/v*) of seeds, with 18% (*w/v*) of soy yoghurt incubated at 30 °C for 4 h 30 min	Pleasant taste and smell. Liquid cream appearance as beverage.	Not necessary.	OK. BPP.
Incubation time of 21 h	Not so nice smell as the previous one: “green” smell; no sweetness. Phase separation, layers with air in between.		

**Table 2:** Results of physico-chemical analysis of raw and toasted *Moringa oleifera* seeds. Values are represented as mean ± standard deviation (*n* = 3). Different letters in samples, per parameter, indicate significant differences between them (*p* < 0.05).

	aw	Moisture (%)	Total Acidity (mEq of acid/L)	Fat (% *w/w*)	Protein (% *w/w*)
**Raw MO seeds**	0.56 ± 0.00	4.74 ± 0.05 ^a^	3.70 ± 0.29	37.24 ± 0.83	36.82 ± 0.20 ^a^
**Toasted MO seeds**	0.48 ± 0.03	0.33 ± 0.05 ^b^	3.50 ± 0.00	34.95 ± 1.34	40.21 ± 0.18 ^b^

**Table 3:** Proximal chemical analysis of *Moringa oleifera*-based beverage (10% *w/v*) and yoghurt-like. The percentage of respective parameters are per 100 mL for MO beverage and for 100 g for MO yoghurt-like. Values are represented as mean ± standard deviation (*n* = 3). * based on raw seed protein content.

	pH	Total Acidity (mEq acid/L)	Fat (%)	Moisture (%)	Ashes (%)	Protein * (%)	Carbohydrate Estim. (%)	Energy (kcal) (kJ)
MO beverage 10% (*w/v*) dry seed	6.03 ± 0.00	10.00 ± 1.32	2.97 ± 0.12	92.67 ± 0.05	0.24 ± 0.05	3.68 ± 0.02	0.44	43.19 ± 0.83 (179.87 ± 3.38)
MO yoghurt-like (4 h 30 min)	5.45 ± 0.02	46.70 ± 2.00	7.25 ± 0.47	81.10 ± 0.43	0.75 ± 0.05	14.73 ± 0.08	0.00	124.13 ± 4.46 (518.51 + 18.38)

**Table 4:** Mineral content of *M. oleifera* seeds (raw and toasted), beverage and yoghurt-like. The mineral contribution of 100 mL of pulse beverage and 100 g of seed or yoghurt, taking into account the dietary reference intakes (DRI) for adults (significant amount of 7.5% for beverage and 15% for seeds and yoghurt) [26] is also presented as a percentage. Values are represented as mean ± SD. Different letters, per mineral element, represent a significant difference between the seed samples (*p* < 0.05).

	Raw MO Seed (mg/100 g)	% DRI	Toasted MO Seed (mg/100 g)	% DRI	MO Beverage (mg/100 mL)	% DRI	MO Yoghurt (mg/100 g)	% DRI
Na	6.82 ± 0.19 ^a^	0.45	9.01 ± 0.78 ^b^	0.60	3.14 ± 0.04	0.21	5.01 ± 0.04	0.33
K	763.49 ± 6.83 ^a^	38.17	747.56 ± 6.38 ^b^	37.38	38.08 ± 0.40	1.90	28.05 ± 0.14	1.40
Ca	139.64 ± 0.76 ^a^	17.45	163.64 ± 1.46 ^b^	20.45	17.86 ± 0.08	2.23	13.29 ± 0.12	1.66
Mg	302.51 ± 2.99 ^a^	80.67	310.47 ± 3.34 ^b^	82.79	29.31 ± 0.04	7.82	15.98 ± 0.15	4.26
P	771.19 ± 3.92 ^a^	110.17	790.68 ± 4.89 ^b^	112.95	72.24 ± 1.01	10.32	40.53 ± 0.19	5.79
S	1994.06 ± 51.42	___	1977.58 ± 23.44	___	156.54 ± 3.90	___	85.88 ± 1.20	___
Fe	9.97 ± 0.07 ^a^	71.18	17.01 ± 0.05 ^b^	121.52	0.50 ± 0.00	3.57	0.26 ± 0.00	1.84
Cu	0.86 ± 0.01 ^a^	85.82	0.91 ± 0.02 ^b^	90.52	0.08 ± 0.00	8.13	0.04 ± 0.00	3.85
Zn	5.20 ± 0.03 ^a^	52.00	6.38 ± 0.02 ^b^	63.81	0.56 ± 0.00	5.61	0.31 ± 0.00	3.09
Mn	1.25 ± 0.01 ^a^	62.35	1.44 ± 0.00 ^b^	72.17	0.15 ± 0.00	7.32	0.08 ± 0.00	3.77
B	0.58 ± 0.01 ^a^	2.88	0.50 ± 0.01 ^b^	2.49	0.00 ± 0.00	___	0.02 ± 0.00	0.11

**Table 5:** The parameters obtained after fitting the flow curves to the Carreau model for all of the beverages and yoghurts are shown (η_0_, zero-shear viscosity; η_∞_, infinite-shear viscosity; and $\dot{\gamma }$c, critical shear rate). Values are represented as mean ± standard deviation (*n* = 3). Same letters in samples per parameter, evidence significant differences between them (*p* < 0.05).

	η_0_ (Pa·s)	η_∞_ (Pa·s)	$\dot{\gamma }$c (s^−1^)
MO yoghurt_4 h 30 min_8 °C	21,465.7 ± 2723.5 ^b,c^	3.3 × 10^−2^ ± 0.3 × 10^−2 a^	6.7 × 10^−4^ ± 0.4 × 10^−4 a^
Low fat dairy yoghurt_8 °C	15,127.2 + 2013.4 ^a,b^	1.4 × 10^−2^ + 0.4 × 10^−2 a,b^	26.6 × 10^−4^ ± 3.0 × 10^−4 a,b^
Soy yoghurt_8 °C	3381.2 ± 277.1 ^a,c^	2.7 × 10^−2^ ± 0.2 × 10^−2 b^	11.0 × 10^−4^ ± 1.9 × 10^−4 b^
Lupin beverage	658.1 ± 34.6 ^d,e^	2.9 × 10^−2^ ± 0.1 × 10^−2 c,d^	2.0 × 10^−4^ ± 0.5 × 10^−4 c^
Chickpea beverage	176.4 ± 22.4 ^d,f^	1.3 × 10^−2^ ± 0.1 × 10^−2 c,e^	5.6 × 10^−4^ ± 1.1 × 10^−4^
MO beverage	17.4 ± 3.1 ^e,f^	0.6 × 10^−2^ ± 0.0 × 10^−2 d,e^	8.1 × 10^−4^ ± 1.9 × 10^−4 c^

## Data Availability

Data is contained within the article.
